# A decrease in NAMPT activity impairs basal PARP-1 activity in cytidine deaminase deficient-cells, independently of NAD^+^

**DOI:** 10.1038/s41598-020-70874-6

**Published:** 2020-08-17

**Authors:** Sandra Cunha Silveira, Géraldine Buhagiar-Labarchède, Rosine Onclercq-Delic, Simon Gemble, Elias Bou Samra, Hamza Mameri, Patricia Duchambon, Christelle Machon, Jérôme Guitton, Mounira Amor-Guéret

**Affiliations:** 1 Institut Curie, UMR 3348, PSL Research University, 91405 Orsay, France; 2 CNRS UMR 3348, Centre Universitaire, 91405 Orsay, France; 3 Université Paris Sud, Université Paris-Saclay, Centre Universitaire, UMR 3348, 91405 Orsay, France; 4 Protein Expression and Purification Core Facility, Institut Curie, PSL Research University, 75248 Paris, France; 5 Université Paris Sud, Université Paris-Saclay, Centre Universitaire, UMR 9187 - INSERM U1196, 91405 Orsay, France; 6 Laboratoire de Biochimie et Toxicologie, Centre Hospitalier Lyon-Sud, Hospices Civils de Lyon, Pierre-Bénite, France; 7 Laboratoire de Chimie Analytique, ISPB, Faculté de Pharmacie, Université Lyon 1, Université de Lyon, Lyon, France; 8 Laboratoire de Toxicologie, ISPB, Faculté de Pharmacie, Université Lyon 1, Université de Lyon, Lyon, France

**Keywords:** Cancer, Cell biology, Molecular biology

## Abstract

Cytidine deaminase (CDA) deficiency causes pyrimidine pool disequilibrium. We previously reported that the excess cellular dC and dCTP resulting from CDA deficiency jeopardizes genome stability, decreasing basal poly(ADP-ribose) polymerase 1 (PARP-1) activity and increasing ultrafine anaphase bridge (UFB) formation. Here, we investigated the mechanism underlying the decrease in PARP-1 activity in CDA-deficient cells. PARP-1 activity is dependent on intracellular NAD^+^ concentration. We therefore hypothesized that defects of the NAD^+^ salvage pathway might result in decreases in PARP-1 activity. We found that the inhibition or depletion of nicotinamide phosphoribosyltransferase (NAMPT), the rate-limiting enzyme in the NAD^+^ salvage biosynthesis pathway, mimicked CDA deficiency, resulting in a decrease in basal PARP-1 activity, regardless of NAD^+^ levels. Furthermore, the expression of exogenous wild-type NAMPT fully restored basal PARP-1 activity and prevented the increase in UFB frequency in CDA-deficient cells. No such effect was observed with the catalytic mutant. Our findings demonstrate that (1) the inhibition of NAMPT activity in CDA-proficient cells lowers basal PARP-1 activity, and (2) the expression of exogenous wild-type NAMPT, but not of the catalytic mutant, fully restores basal PARP-1 activity in CDA-deficient cells; these results strongly suggest that basal PARP-1 activity in CDA-deficient cells decreases due to a reduction of NAMPT activity.

## Introduction

The maintenance of genome stability is crucial for preventing various aging-related diseases, including cancer^1^. We have shown that cytidine deaminase (CDA) plays an essential role in maintaining genome integrity. CDA is an enzyme of the pyrimidine salvage pathway catalyzing the hydrolytic deamination of cytidine (C) and deoxycytidine (dC) to uridine (U) and deoxyuridine (dU), respectively^2^.

CDA activity has been widely studied in the context of the inactivation of nucleoside analogs widely used in chemotherapy. The determination of CDA expression status within cancer cells and tissues is opening up new possibilities for cancer treatment^3–5^. We have shown that CDA expression is downregulated in about 60% of cancer cells and tissues, mostly due to DNA methylation^3^. We have also demonstrated the existence of a causal link between the expression of CDA and other genes, in particular the gene encoding the microtubule-associated protein Tau (MAPT), highlighting the importance of analyzing CDA expression levels alone or together with the expression of other genes, as a relevant and predictive marker of susceptibility to antitumor drugs^3,6^. The pyrimidine pool disequilibrium resulting from CDA deficiency also contributes to the genetic instability of cells, particularly in Bloom syndrome (BS)^7^. Indeed, we have reported that the resulting intracellular accumulation of dC and dCTP decreases PARP-1 activity in basal conditions and in response to genotoxic stress, leading to the accumulation of unreplicated DNA during mitosis and, thus, to high levels of UFB formation^8–10^. However, based on in vitro data that were not consistent with the direct inhibition of PARP-1 by dC or dCTP in vivo, we hypothesized that the intracellular accumulation of dC and dCTP might impair PARP-1 activity indirectly^10^. We therefore investigated the mechanism responsible for decreasing basal PARP-1 activity in CDA-deficient cells. PARP-1 is a multifunctional enzyme that mediates several aspects of the DNA damage response through its poly(ADP-ribosyl)ation (PARylation) activity, involving the transfer of PAR units from nicotinamide-adenine-dinucleotide (NAD^+^) to diverse acceptor proteins, including histones^11^. Thus, PARP-1 plays a key role in preventing genetic instability, and its activity is dependent on intracellular NAD^+^ concentration. NAD^+^ is mostly synthesized from the precursor nicotinamide (NAM), via the salvage pathway. NAMPT is the rate-limiting enzyme in NAD^+^ biosynthesis via this pathway^12,13^. It catalyzes the transfer of a phosphoribosyl group from 5-phosphoribosyl-pyrophosphate (PRPP) to NAM, generating the NAD^+^ intermediate nicotinamide mononucleotide (NMN), which is converted to NAD^+^ by nicotinamide mononucleotide adenylyltransferase (NMNAT)^14,15^. Through this function in NAD^+^ biosynthesis, NAMPT activity is crucial for regulation of the activity of NAD^+^-dependent enzymes, such as sirtuins and PARPs^16^.

In this study, we investigated the mechanism responsible for decreasing basal PARP-1 activity in CDA-deficient cells. We found that NAMPT inhibition or depletion reproduced the cellular phenotype associated with CDA deficiency, lowering basal PARP-1 activity and increasing UFB frequency to levels similar to those in CDA-deficient cells. We also showed that the decrease in basal PARP-1 activity was independent of NAD^+^ levels. Finally, we demonstrated that the expression of exogenous wild-type NAMPT fully restored basal PARP-1 activity, thereby preventing the increase in UFB frequency in CDA-deficient cells, whereas no such effect was observed with the catalytic mutant. These results provide the first evidence of a link between the pyrimidine and NAD^+^ salvage pathways.

## Results

### NAMPT inhibition decreases basal PARP-1 activity, mimicking CDA deficiency

**Figure 1:**
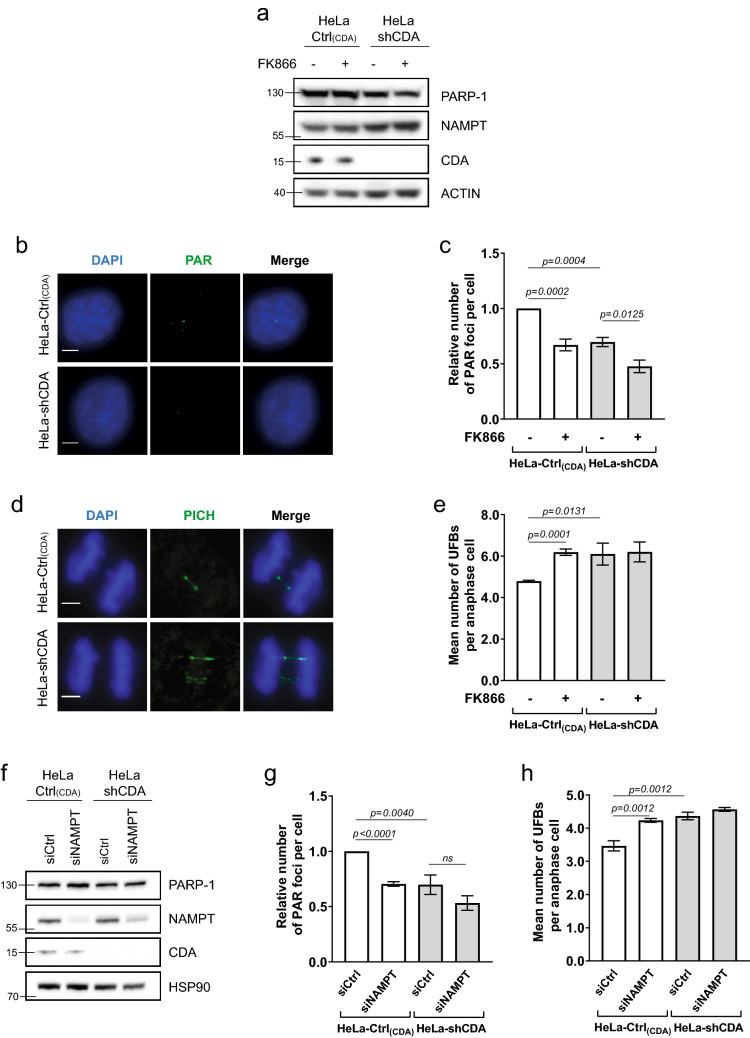
NAMPT inhibition or depletion impairs basal PARP-1 activity. (**a**) PARP-1, NAMPT and CDA protein levels assessed by immunoblotting in HeLa-Ctrl_(CDA)_ and HeLa-shCDA cell lines left untreated or treated with 1 μM FK866 for 10 h. Actin was used as protein loading control. (**b**) Representative immunofluorescence deconvoluted *z*-projection images of HeLa-Ctrl_(CDA)_ and HeLa-shCDA cells showing PAR foci in interphase cells. Scale bar: 5 µm. (**c**) Analysis of PAR foci number in HeLa-Ctrl_(CDA)_ and HeLa-shCDA cell lines left untreated or treated with 1 μM FK866 for 10 h. The data shown are the means ± SD from four independent experiments (> 350 cells per condition). (**d**) Representative immunofluorescence deconvoluted *z*-projection images of PICH-positive UFBs in HeLa-Ctrl_(CDA)_ and HeLa-shCDA anaphase cells. DNA was visualized by DAPI staining (blue). UFBs were stained with PICH antibody (in green, Alexa Fluor 555). Scale bar: 5 µm. (**e**) Mean number of UFBs per anaphase cell, for HeLa-Ctrl_(CDA)_ and HeLa-shCDA cell lines left untreated or treated with 1 μM FK866 for 10 h. The data shown are the means $\pm$ SD from three independent experiments (> 80 anaphase cells per condition). (**f**) PARP-1, NAMPT and CDA protein levels assessed by immunoblotting in HeLa-Ctrl_(CDA)_ and HeLa-shCDA cell lines transiently transfected with the indicated siRNAs twice successively for a total of 144 h (96 h + 48 h). HSP90 was used as protein loading control. (**g**) Analysis of PAR foci number in HeLa-Ctrl_(CDA)_ and HeLa-shCDA cell lines transiently transfected with the indicated siRNAs. The data shown are the means ± SD from four independent experiments (> 350 cells per condition). (**h**) Mean number of UFBs per anaphase cell, for HeLa-Ctrl_(CDA)_ and HeLa-shCDA cell lines transiently transfected with the indicated siRNAs. The data shown are means ± SD from three independent experiments (> 120 anaphase cells per condition). The significance of differences was assessed with Student’s *t*-test.

We explored the potential link between the decrease in basal PARP-1 activity in CDA-deficient cells and NAD^+^ metabolism, by investigating the effect of inhibiting NAMPT activity on basal PARP-1 activity. We used two pairs of isogenic cellular models of CDA deficiency displaying decreases in basal PARP-1 activity: the GM8505B-derived BS cell line, which has a mutated *BLM* gene and strongly downregulated CDA expression (BS-Ctrl_(BLM)_; BLM^-^/CDA^-^), and its counterpart stably expressing an exogenous GFP-BLM construct restoring the expression of both BLM and CDA (BS-BLM; BLM^+^/CDA^+^); and a HeLa cell line stably expressing an adenoviral short hairpin RNA (shRNA) specific for CDA, displaying strong CDA downregulation (HeLa-shCDA: BLM^+^/CDA^-^), and its control counterpart expressing CDA (HeLa-Ctrl_(CDA)_; BLM^+^/CDA^+^)^7,10^. As expected, both CDA-deficient cell lines contained significantly larger amounts of cytidine and smaller amounts of uridine than their control counterparts (Figure S1a and S1b). We treated the cells with the NAMPT inhibitor FK866. FK866 treatment did not affect the levels of the PARP-1, NAMPT or CDA proteins (Fig. 1a and S1c), and did not lead to an inhibition of recombinant PARP-1 protein activity (Figure S1d). We then performed immunofluorescence assays to assess the basal cellular levels of PARylation, by measuring the relative number of PAR foci as readout of cellular PARP-1 activity (Fig. 1b). FK866 treatment significantly decreased the prevalence of PAR foci in both CDA-proficient HeLa and BS-BLM cells, to the levels observed in CDA-deficient cells (Figs. 1c and S1e). FK866 treatment also decreased significantly the frequency of PAR foci in both cell lines lacking CDA (Figs. 1c and S1e). We previously reported that decreases in basal PARP-1 activity lead to an increase in the frequency of UFB formation^10^. We therefore analyzed the frequency of UFBs in these cells, by staining them with antibodies specific for the helicase-like protein PICH (Plk1-interaction checkpoint “helicase”). This is the only way to detect the total UFB population^17^ (Fig. 1d). FK866 treatment increased UFB frequency in CDA-expressing cells to levels similar to those in CDA-deficient cells, but had no effect on UFB frequency in CDA-deficient cells (Figs. 1e and S1f.).

For confirmation of these results, we induced a transient depletion of NAMPT in the two pairs of isogenic cell lines, by transfecting them with a pool of four NAMPT-targeting siRNAs. We then assessed changes in NAMPT protein levels (Figs. 1f and S1g) and in the frequencies of PAR foci and UFBs (Figs. 1g, h, S1h and S1i). NAMPT depletion had no impact on PAR foci or UFB frequencies in CDA-deficient cells (Figs. 1g,h, S1h and S1i). However, it decreased PAR focus frequency (Fig. 1g and S1h) and increased UFB frequency (Figs. 1h and S1i) in CDA-proficient cells, to levels similar to those observed in CDA-deficient cells. Thus, NAMPT inhibition or depletion in CDA-proficient cells decreases the basal activity of PARP-1 and increases the frequency of UFBs to levels similar to those in CDA-deficient cells, mimicking CDA deficiency. Moreover, no additive effect of CDA deficiency and NAMPT inhibition or depletion was observed on UFB frequency, suggesting that CDA and NAMPT probably prevent UFB formation by acting on the same pathway.

### The decrease in basal PARP-1 activity resulting from NAMPT inhibition is independent of NAM accumulation and NAD^+^ levels

**Figure 2:**
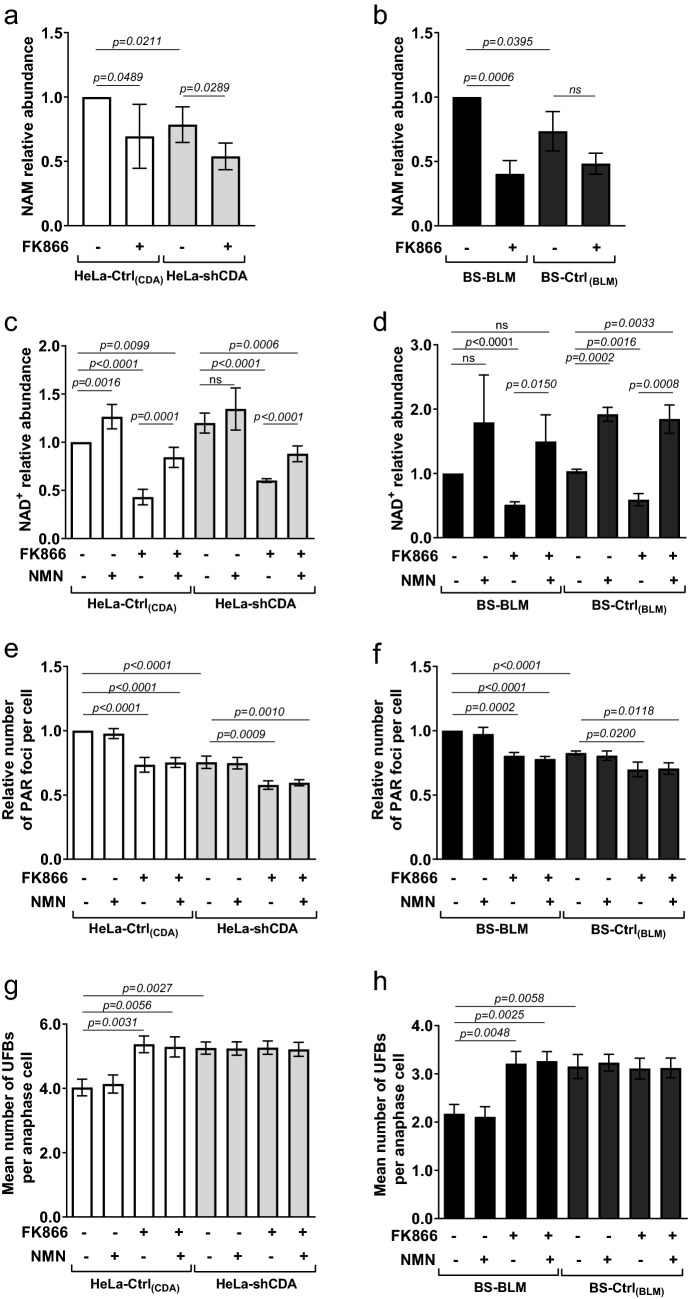
The decrease in basal PARP-1 activity resulting from NAMPT inhibition is independent of NAM accumulation and NAD^+^ levels. (**a**) and (**b**) Analysis of intracellular NAM levels in HeLa-Ctrl_(CDA)_ and HeLa-shCDA cell lines (**a**) or in BS-BLM and BS-Ctrl_(BLM)_ cell lines (**b**) left untreated or treated with 1 μM FK866 for 10 h. The data shown are means ± SD from four (**a**) or three (**b**) independent experiments, respectively. (**c**) and (**d**) Analysis of intracellular NAD^+^ levels (luciferase assay) in HeLa-Ctrl_(CDA)_ and HeLa-shCDA cell lines (**c**) or in BS-BLM and BS-Ctrl_(BLM)_ (**d**) left untreated or treated with 1 μM FK866 for 10 h and/or with 500 μM NMN for 24 h. The data shown are means ± SD from five (**c**) or from three (**d**) independent experiments, respectively. (**e**) and (**f**) Analysis of PAR foci number in HeLa-Ctrl_(CDA)_ and HeLa-shCDA cell lines (**e**) or in BS-BLM and BS-Ctrl_(BLM)_ cell lines (**f**) left untreated or treated with 1 μM FK866 for 10 h and/or with 500 μM NMN for 24 h. The data shown are means ± SD from four independent experiments (> 400 cells per condition) (**e**) or from three independent experiments (> 400 cells per condition) (**f**), respectively. (**g**) and (**h**) Mean number of UFBs per anaphase cell, for HeLa-Ctrl_(CDA)_ and HeLa-shCDA cell lines (**g**) or for BS-BLM and BS-Ctrl_(BLM)_ cell lines (**h**) left untreated or treated with 1 μM FK866 for 10 h and/or with 500 μM NMN for 24 h. The data shown are means ± SD from three independent experiments (> 100 anaphase cells per condition) (**g**) or from three independent experiments (> 120 anaphase cells per condition) (**h**). The significance of differences was assessed with Student’s *t*-test.

As NAMPT converts NAM to NMN, which is in turn converted to NAD^+^ by NMNAT (Figure S2a), NAMPT defects would be expected to result in higher NAM and lower NAD^+^ levels. We then analyzed NAM levels by LC-HRMS (liquid chromatography-high-resolution mass spectrometry) in cells left untreated or treated with FK866. Surprisingly, FK866 treatment did not increase NAM levels in any of the four cell lines. Moreover, NAM levels were slightly but significantly lower in CDA-deficient cells than in control cells (Fig. 2a and b), disabling us to conclude about the possible contribution of unbalanced NAM levels to the decrease in basal PARP-1 activity in CDA-deficient cells.

We then analyzed NAD^+^ levels by LC-HRMS or with a luciferase assay. NAD^+^ levels were significantly higher in CDA-depleted HeLa cells than in control cells, whereas no significant difference was observed between the BS-Ctrl_(BLM)_ and BS-BLM cell lines, suggesting that the decrease in PARP-1 activity in CDA-deficient cells was independent of NAD^+^ levels (Figures S2b, S2c, S2d and S2e).

For confirmation of these results, we evaluated NAD^+^ levels, PAR foci and UFB frequencies after treatment with NMN and/or FK866 (Figure S2a). We observed either no change or a significant increase in NAD^+^ levels in the four cell lines in response to NMN treatment (Fig. 2c,d). Several studies have reported that the blockade of NAD^+^ resynthesis by inhibition of the rate-limiting enzyme NAMPT can be circumvented by alternative metabolic pathways leading to NAD^+^ formation^18,19^. Thus, these results strongly suggest that, in the context of CDA deficiency, the partial inhibition of NAMPT is circumvented for NAD^+^ synthesis.

By contrast, NAD^+^ levels were significantly lower in these cells after FK866 treatment, as expected (Fig. 2c,d). Moreover, NMN treatment partly rescued NAD^+^ levels in the four FK866-treated cell lines (Fig. 2c,d). Importantly, although the addition of NMN made it possible to maintain high levels of NAD^+^ in cells treated with FK866, it did not prevent the decrease in PAR focus frequency and the subsequent increase in UFB frequency resulting from NAMPT inhibition by FK866 in CDA-proficient HeLa-Ctrl_(CDA)_ or BS-BLM cells (Fig. 2e–h). Together, these results indicate that NAD^+^ levels do not reflect NAMPT activity in the context of CDA deficiency, and demonstrate that the decrease in basal PARP-1 activity resulting from NAMPT inhibition is completely independent of cellular NAD^+^ levels.

### The decrease in basal PARP-1 activity in CDA-deficient cells is rescued by the expression of an exogenous wild-type NAMPT, but not by a mutated NAMPT

**Figure 3:**
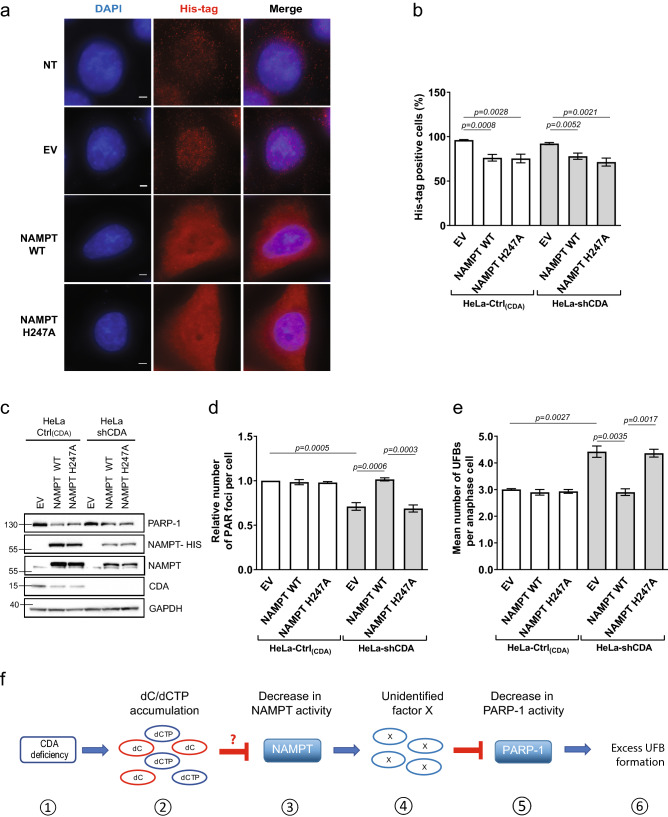
The low levels of PARP-1 activity in CDA-deficient cells are rescued by the overexpression of wild-type NAMPT (**a**) Representative immunofluorescence deconvoluted *z*-projection images showing DAPI and His-tag staining in HeLa-Ctrl_(CDA)_ cells not transfected (NT) or transiently transfected with pPM-C-His empty vector (EV), or with a pPM-C-His construct expressing wild-type NAMPT (NAMPT WT) or mutated NAMPT (NAMPT H247A). Nuclei were visualized by DAPI staining (blue) and the His-tag was visualized with Alexa Fluor 555 (red). Scale bar: 5 µm. (**b**) Percentage of His-tag-positive cells among HeLa-Ctrl_(CDA)_ and HeLa-shCDA cells transiently transfected with EV, NAMPT WT or NAMPT H247A. The data shown are means ± SD from four independent experiments. (**c**) PARP-1, NAMPT, NAMPT-HIS and CDA proteins levels assessed by immunoblotting in HeLa-Ctrl_(CDA)_ and HeLa-shCDA cells transiently transfected with EV, NAMPT WT or NAMPT H247A. (**d**) Relative number of PAR foci in HeLa-Ctrl_(CDA)_ and HeLa-shCDA) cells transiently transfected with EV, NAMPT WT or NAMPT H247A. The data shown are means ± SD from four independent experiments (> 800 cells per condition). (**e**) Mean number of UFBs per anaphase cell, for HeLa-Ctrl_(CDA)_ and HeLa-shCDA cell lines transiently transfected with EV, NAMPT WT or NAMPT H247A. Error bars represent means ± SD from three independent experiments (> 120 anaphase cells per condition). The significance of differences was assessed in Student’s *t*-tests. (**f**) (1) CDA deficiency leads to (2) intracellular dC/dCTP accumulation that (3) decreases NAMPT activity, directly or indirectly, leading to the (4) intracellular accumulation of an as yet unidentified factor X (5) lowering basal PARP-1 activity, causing (6) excess UFB formation.

For formal confirmation of the involvement of the decrease in NAMPT enzymatic activity in the low basal levels of PARP-1 activity observed in CDA-deficient cells, we investigated whether overexpressing an exogenous NAMPT could restore the levels of PARP-1 activity in CDA-deficient cells. We used a construct expressing either a wild-type (WT) NAMPT or the NAMPT protein with a mutated catalytic site (H247A)^20^. In parallel, two recombinant proteins — WT and H247A-mutated NAMPT proteins—were produced and their activity was assessed by LC-HRMS or in an assay measuring the conversion of ^14^C^-^NAM to ^14^C-NMN. The wild-type NAMPT was highly active, whereas the mutated protein had no activity, as expected (Figures S3a and S3b). Similar numbers of CDA-deficient and CDA-proficient HeLa cells were transfected with the wild-type and mutated NAMPT constructs (Fig. 3a–c). The levels of exogenous wild-type and mutated NAMPT proteins (NAMPT-His and the upper NAMPT band) expressed were lower in CDA-deficient cells than in control cells, and transfection with any NAMPT-expressing construct decreased PARP-1 and CDA protein levels (Fig. 3c). However, the expression of exogenous wild-type or mutated NAMPT had no effect on the frequencies of PAR foci or UFBs in CDA-proficient HeLa cells, whereas the expression of wild-type NAMPT, but not of the mutated NAMPT, fully restored the frequency of PAR foci and, consequently, UFB frequency to normal levels in CDA-deficient HeLa cells (Fig. 3d,e). Similarly, the expression of exogenous wild-type NAMPT in CDA-deficient BS cells led to a significant increase in the frequency of PAR foci and a significant decrease in UFB frequency (Figures S3c, S3d and S3e), whereas the mutated NAMPT had no such effect. Thus, basal PARP-1 activity was fully restored in both BS and CDA-deficient HeLa cells by the expression of an exogenous wild-type NAMPT, whereas the inactive NAMPT mutant had no effect in these cells. These data indicate that the lower basal PARP-1 activity in CDA-deficient cells results from a lower level of NAMPT activity in these cells.

## Discussion

We report here, for the first time, an unexpected link between CDA and NAMPT, CDA deficiency probably being associated with a decrease in NAMPT activity. Indeed, we found that NAMPT inhibition or depletion in CDA-expressing cells reproduced the main feature of CDA deficiency: a decrease in basal PARP-1 activity leading to an increase in UFB frequency.

We previously showed that the pyrimidine pool disequilibrium resulting from CDA deficiency decreases basal PARP-1 activity, leading to UFB formation. Increasing the size of the intracellular dC and dCTP pools by culturing CDA-expressing cells in the presence of dC was sufficient to decrease PARP-1 activity. However, our in vitro data were not consistent with the direct inhibition of PARP-1 by dC or dCTP^10^. We report here that the inhibition or depletion of NAMPT activity decreases PARP-1 activity. These findings strongly suggest that the lower levels of basal PARP-1 activity in CDA-deficient cells result from the partial inhibition of NAMPT activity by the excess dC and dCTP. We tested this hypothesis by performing several experiments with cell extracts prepared under various conditions, or by studying the recombinant NAMPT protein in vitro in the presence of various concentrations of dC or dCTP, with an assay measuring the conversion of ^14^C^-^NAM to ^14^C-NMN, or with an LC-HRMS approach for the selective detection of NAM and NMN. Neither of these experimental approaches yielded reproducible results and, despite our best efforts, we were unable to identify the cause of this lack of reproducibility. It probably resulted, at least in part, from the unusually high affinity of NAMPT for NAM^21^, making it very difficult to detect its inhibition by less effective substrates. We cannot, therefore, exclude the possibility that the decrease in NAMPT activity was a direct or indirect consequence of the excess cellular dC and/or dCTP.

Another hypothesis based on our results showing that NAMPT inhibition leads to a decrease in basal PARP-1 activity independently of NAD^+^ levels is that the excess NAM resulting from NAMPT inhibition inhibits PARP-1. However, we detected no excess of NAM in CDA-deficient cells. Indeed, we found a small but significant decrease in NAM levels in these cells. Excess NAM that is not recycled is known to be methylated by nicotinamide N-methyltransferase to generate MNAM (methylated NAM) for excretion from the body^21,22^. However, some of the excess NAM may be captured by PARP-1 in CDA-deficient cells, before NAM clearance, decreasing its activity.

We propose a model in which an as yet unidentified factor resulting from the decrease in NAMPT activity impairs PARP-1 activity (Fig. 3f).

Our data also reveal that cellular NAD^+^ levels were unchanged or increased in CDA-deficient cells, and that the decrease in PARP-1 activity resulting from the inhibition of NAMPT activity was independent of cellular NAD^+^ levels. These results indicate that NAD^+^ levels are maintained in cells, despite NAMPT inhibition, suggesting that, in CDA-deficient cells, the partial inhibition of NAMPT is circumvented for NAD^+^ synthesis, as proposed to explain inconclusive attempts to reduce NAD^+^ production in patients with cancer^18,19^. This point is of particular interest, because cancer cells require high levels of NAD^+^, and the mechanisms underlying the maintenance of these high levels may be involved in resistance to anticancer treatments, particularly those targeting NAMPT^23^. Activation of the de novo NAD^+^ biosynthesis pathway by the upregulation of quinolinate phosphoribosyl transferase (QPRT) has been reported to confer resistance to NAMPT inhibition^23^. However, PARP-1 activity is partially inhibited in CDA-deficient cells, and would therefore be expected to consume less NAD^+^, thereby contributing to the maintenance of cellular NAD^+^ levels.

In conclusion, our results are consistent with a decrease in NAMPT activity in CDA-deficient cells, leading to a decrease in basal PARP-1 activity. In several preclinical models, combinations of NAMPT and PARP-1 inhibitors have been shown to induce cancer cell death and tumor regression, revealing a synthetic lethal interaction between NAMPT and PARP-1 deficiencies^24–26^. Our study suggests that the concomitant reduction of NAMPT and PARP-1 activities in CDA-deficient tumor cells may be associated with a better prognosis.

## Materials and methods

### Cell culture and drug treatments

BS GM08505B and HeLa cells were purchased from the Coriell Institute and ATCC, respectively. Cell lines were cultured in Dulbecco’s modified Eagle’s medium (DMEM) supplemented with 10% FCS.

BS-Ctrl_(BLM)_ and BS-BLM cells, and HeLa-Ctrl_(CDA)_ and HeLa-shCDA cells, were obtained as previously reported^7,10^

All cell lines were routinely checked for mycoplasma infection. Authenticity was assessed by comparing the short tandem repeat profile generated with the profiles present in the Deutsche Sammlung von Mikroorganismen und Zellkulturen.

Nicotinamide mononucleotide was purchased from Sigma (NMN #N3501), and FK866 was purchased from Calbiochem (#481908). Drugs were added to the cell culture medium at the following concentrations and for the following amounts of time: FK866, 1 µM for 10 h; and NMN, 500 µM for 24 h.

### Transfection with siRNA

Cells were transfected with a pool of four siRNAs specific for NAMPT (ON-TARGETplus SMART-pool, Dharmacon) or negative control siRNAs (ON-TARGETplus siCONTROL Non Targeting Pool, Dharmacon), in the presence of DharmaFECT 1 (Dharmacon). We used a standard siRNA concentration of 100 nM. The sequences of the siRNAs are provided in Supplementary Table 1.

### NAD^+^ quantification in a luciferase assay

NAD^+^ levels were quantified with a luciferase assay provided in the NAD^+^/NADH Glo Assay kit (#G9071, Promega), used according to the manufacturer’s instructions. NAD^+^ levels were normalized against cell viability in a CellTiter-Glo Luminescent Cell Viability Assay (#G7570, Promega), according to the manufacturer’s instructions. Luminescence was read on a Tristar2 multimode microplate reader (Berthold Technologies).

### Quantification of intracellular NAD^+^, NAM, NMN, cytidine and uridine by LC-HRMS

Intracellular NAM, NMN, NAD^+^, cytidine and uridine were extracted with a mixture of cold methanol/water/formic acid (70/27/3, v/v/v). We added ^13^C_6_NAM, thioNAD and ^13^C_5_Uridine as internal standards, and the supernatant of the extract was evaporated off to dryness. The residue was then resuspended in 200 µl of mobile phase and 10 µl was injected into the HPLC-HRMS system. Chromatographic separation was achieved on a Ultimate 3,000 HPLC system (Thermo) with an Atlantis dC 18 Waters column (150 × 2.1 mm, 3 µm) and a gradient from 0.1% formic acid in water to 0.1% formic acid in methanol. NAM, NMN, NAD^+^, cytidine, uridine and thioNAD were detected with a HRMS Q Exactive Plus mass spectrometer (Thermo), after positive or negative electrospray ionization. The m/z values in positive mode were 123.05528, 335.06390, 664.11639, 129.07542, 680.09355 and 244.09280 for NAM, NMN, NAD^+^, ^13^C_6_NAM, thioNAD and cytidine, respectively. The values of m/z in negative mode were 243.06226 and 248.07903 for uridine and ^13^C_5_uridine, respectively. Results are expressed as (NAM area)/(^13^C_6_NAM area), (NAD^+^ area)/(thioNAD area), (cytidine area)/(^13^C_5_uridine area) and (uridine area)/(^13^C_5_uridine area) ratios normalized according to the number of cells.

### Immunoblotting

Immunoblotting was performed as previously reported^7,10^. The following antibodies were used for detection: rabbit anti-NAMPT (#A300-372A, Bethyl Laboratories, Inc., 1:20,000), rabbit anti-PARP-1 (#ALX-210-302-R100, Enzo Life Sciences, 1:4000), rabbit anti-CDA (#ab56053, Abcam, dilution 1:500), rabbit anti-BLM (#ab2179, Abcam, dilution 1:5000), rabbit anti-β-actin (#A2066, Sigma-Aldrich, 1:5000), rabbit anti-HSP90 (#ab2928, Abcam, 1:5000), rabbit anti-His-tag (#66,005-I, Proteintech, dilution 1:1000), mouse anti-GAPDH (#G8795 1:1000), horseradish peroxidase (HRP)-conjugated goat anti-rabbit IgG (#A9169, Sigma-Aldrich, 1:5000), and HRP-conjugated goat anti-mouse IgG (#A3682, Sigma-Aldrich, 1:5000). Bands were visualized by chemiluminescence (Clarity Western ECL Substrate, Bio-Rad), with a ChemiDoc XRS+ Molecular Imager and Image Lab Software (Bio-Rad).

### Immunofluorescence microscopy

Immunofluorescence staining and analysis were performed as previously described^27^. Primary and secondary antibodies were used at the following concentrations: rabbit anti-PICH antibody (H00054821-D01P, Abnova, dilution 1:150), and goat anti-rabbit Alexa Fluor 555 (#A21429, Life Technologies, dilution 1:500). Cell images were acquired with a 3-D deconvolution imaging system consisting of a Leica DM RXA microscope equipped with a piezoelectric translator (PIFOC; PI) placed at the base of a 63× PlanApo N.A. 1.4 objective, and a CoolSNAP HQ interline CCD camera (Photometrics). Stacks of conventional fluorescence images were collected automatically at a *Z*-distance of 0.2 micrometer (Metamorph software; Molecular Devices). NH_4_Cl incubation was not performed for His-tag staining. The primary and secondary antibodies for His-tag staining were used at the following concentrations: rabbit anti-His-tag (#66005-I, Proteintech, dilution 1:500), and goat anti-mouse Alexa Fluor 555 (#A21050, Life Technologies, dilution 1:500).

### Poly(ADP)-ribose immunofluorescence

Poly(ADP)-ribose immunofluorescence was analyzed as previously described^10^.

### Colorimetric PARP assay kit candidate inhibitor screening

PARP-1 inhibition by FK866 was assessed with the HT universal colorimetric PARP assay kit, with histone-coated strip wells (4677-096-K from Trevigen), according to the manufacturer’s instructions.

### Cloning, site-directed mutagenesis and vector transfection

Site-directed mutagenesis was performed with the pPM-C-His vector carrying the full-length cDNA for human NAMPT (#314140210600, Applied Biological Materials, abmgood) (GenBank accession no. BC072439). The His codon in position 247 of NAMPT was mutated into an Ala (H27A) codon with the QuikChange XL site-directed mutagenesis kit (#200251 Agilent), according to the manufacturer’s instructions. The primer sequences are provided in Supplementary Table 2. Cells were transfected by incubation with NAMPT WT, NAMPT H247A or empty vector in the presence of JetPrime (Polyplus Transfection) for 48 h.

### Protein production and purification

A pPM-C-His plasmid carrying the full-length cDNA for human NAMPT was purchased from Applied Biological Materials (abmgood). The NAMPT cDNA was amplified by PCR with primers containing *Nhe*I and *Xho*I sites. The amplified DNA fragment was inserted between the *Nhe*I and *Xho*I sites of pET24a(+)(#69749–3), Novagen), resulting in the attachment of a his_6_ tag at the C-terminus of the protein. The H247A mutation was generated with the QuikChange XL site-directed mutagenesis kit (#200251, Agilent) according to the manufacturer’s instructions. The primer sequences are provided in Supplementary Table 2. Both constructs were overexpressed in BL21 Dsbc plSO in 1 L of Terrific Broth. Expression was induced by incubation with 0.5% arabinose and 1 mM IPTG at 20 °C overnight, with shaking at 160 rpm. The cells were harvested by centrifugation at 4000×*g* for 15 min at 4 °C.

The cell pellets were suspended in lysis buffer (20 mM Tris–HCl pH 8, 500 mM NaCl, 10% glycerol, 1 mM TCEP and 1× Complete EDTA-free protease inhibitor cocktail (Roche)). The cell suspension was disrupted by passage through a T75 cell disruptor (Constant Systems). The resulting cell lysate was centrifuged at 43,000×*g* for 1 h at 4 °C. The supernatant was applied to a HisTrap HP column (GE Healthcare), washed thoroughly and the proteins were eluted in elution buffer (20 mM Tris–HCl pH 8, 500 mM NaCl, 10% glycerol, 0.5 mM TCEP, 400 mM imidazole). After overnight dialysis against 20 mM Tris–HCl pH 8, 100 mM NaCl, 10% glycerol, 0.5 mM TCEP, the eluate was loaded onto a Capto Q ImpRes ion exchange column (GE Healthcare) for elution with a continuous gradient NaCl (0.1–1 M) in the same buffer. Fractions containing NAMPT were dialyzed against 20 mM Tris–HCl pH 8, 500 mM NaCl, 10% glycerol, 0.5 mM TCEP, and loaded on a HisTrap HP column. NAMPT was eluted with an imidazole gradient. NAMPT-containing fractions were pooled and dialyzed against 20 mM Tris–HCl pH 8, 100 mM NaCl, 10% glycerol, 0.5 mM TCEP. The protein was visualized by SDS-PAGE in a 4–20% acrylamide gel and protein concentration was determined by measuring absorbance at 280 nm. The mutant protein was purified with the same protocol as the wild-type protein.

### NAMPT enzyme assay measuring the conversion of ^14^C^-^NAM to ^14^C-NMN

NAMPT enzymatic activity was measured as previously described^28^. We diluted 1 µg of wild-type or mutated recombinant NAMPTs in assay buffer without substrate (10 µl in total), which was then mixed with 50 µl reaction mixture (20 mmol/l Tris–HCl pH7.4; 2.5 mmol/l ATP; 50 mmol/l NaCl; 12,5 mmol/l MgCl_2_; 2 mmol/l DTT; 0.5 mmol/l PRPP; 13 µmol/l ^14^C-nicotinamide) and incubated at 37 °C for 1 h. Each point was tested in triplicate. The reaction was stopped by adding 1.8 ml acetone to each tube. The mixture was then pipetted onto acetone-presoaked glass microfiber filters (GF/A diameter 24 mm). The filters were rinsed twice, with 2 ml acetone each, air-dried and transferred to vials with 2 ml scintillation cocktail. We quantified ^14^C-NMN radioactivity in a liquid scintillation counter, as the number of disintegrations per minute (dpm), with a Tri-Carb 2910 TR machine (PerkinElmer). We subtracted the values for the buffer as background, and the mean number of dpm obtained per 1 µg of enzyme was compared. The histogram shows the normalized results for wt and mutated NAMPT activities. Each experiment was performed three times.

### NAMPT enzyme assay by HPLC-HRMS

NAMPT activity was studied by monitoring the decrease in NAM levels following incubation in a mixture with a final volume of 60 µL. The incubation mixture contained Tris–HCl (pH7.4, 17 mM), ATP (2.1 mM), NaCl (83 mM), MgCl_2_ (10.5 mM), DTT (2 mM) and PRPP (0.2 mM). Preincubation with NAMPT (0.5 µg) was performed for 10 min at 37 °C. For some samples, dCTP (10 µM) was added at the same time as NAMPT. We then added NAM (5 µM) and incubated for 45 min, after which, 25 µl of the mixture was transferred to 300 µl of a cold methanol/water mixture (70/30, v/v) to stop the reaction. ^13^C_6_NAM was added as an internal standard, and the supernatant of the extract was evaporated to dryness. The residue was resuspended in 200 µl of mobile phase and 10 µl was injected into the HPLC-HRMS system. Chromatographic separation and mass spectrometry detection were performed as described above for the determination of intracellular NAM, NMN and NAD^+^.

### Statistical analysis

At least three independent experiments were carried out to generate each data set. The statistical significance of differences was calculated with two-tailed unpaired Student’s *t*-tests.

## Supplementary information

Supplementary file1.
