# Supplementary material for: A decrease in NAMPT activity impairs basal PARP-1 activity in cytidine deaminase deficient-cells, independently of NAD+
Source: Sci Rep. 2020 Aug 17;10:13907. doi: 10.1038/s41598-020-70874-6 (PMC7431583; doi:10.1038/s41598-020-70874-6)
Supplement: Supplementary file 1 — Supplementary file1. [file 41598_2020_70874_MOESM1_ESM.pdf]

Supplementary Information

A decrease in NAMPT activity impairs basal PARP-1 activity in cytidine deaminase deficient-cells, independently of NAD^+

Sandra Cunha Silveira[1,2,3,9], Géraldine Buhagiar-Labarchède[1,2,3,9], Rosine Onclercq-Delic[1,2,3,9], Simon Gemble[1,2,3], Elias Bou Samra[1,2,3], Hamza Mameri[1,2,3], Patricia Duchambon[4,5], Christelle Machon[6,7], Jérôme Guitton[6,8], Mounira Amor-Guéret[1,2,3,10*].

[1]Institut Curie, PSL Research University, UMR 3348, 91405, Orsay, France

[2]CNRS UMR 3348, Centre Universitaire, 91405, Orsay, France

[3]Université Paris Sud, Université Paris-Saclay, Centre Universitaire, UMR 3348, 91405, Orsay, France

[4]Protein Expression and Purification Core Facility, Institut Curie, PSL Research University, 75248 Paris, France

[5]Université Paris Sud, Université Paris-Saclay, Centre Universitaire UMR 9187 - INSERM U1196, 91405 Orsay, France.

[6]Laboratoire de Biochimie et Toxicologie, Centre Hospitalier Lyon-Sud, Hospices Civils de Lyon, Pierre-Bénite, France

[7]Laboratoire de Chimie Analytique, ISPB, Faculté de Pharmacie, Université Lyon 1, Université de Lyon, Lyon, France

[8]Laboratoire de Toxicologie, ISPB, Faculté de Pharmacie, Université Lyon 1, Université de Lyon, Lyon, France

[9]These authors contributed equally

[10]Lead contact

[*]Correspondence: mounira.amor@curie.fr

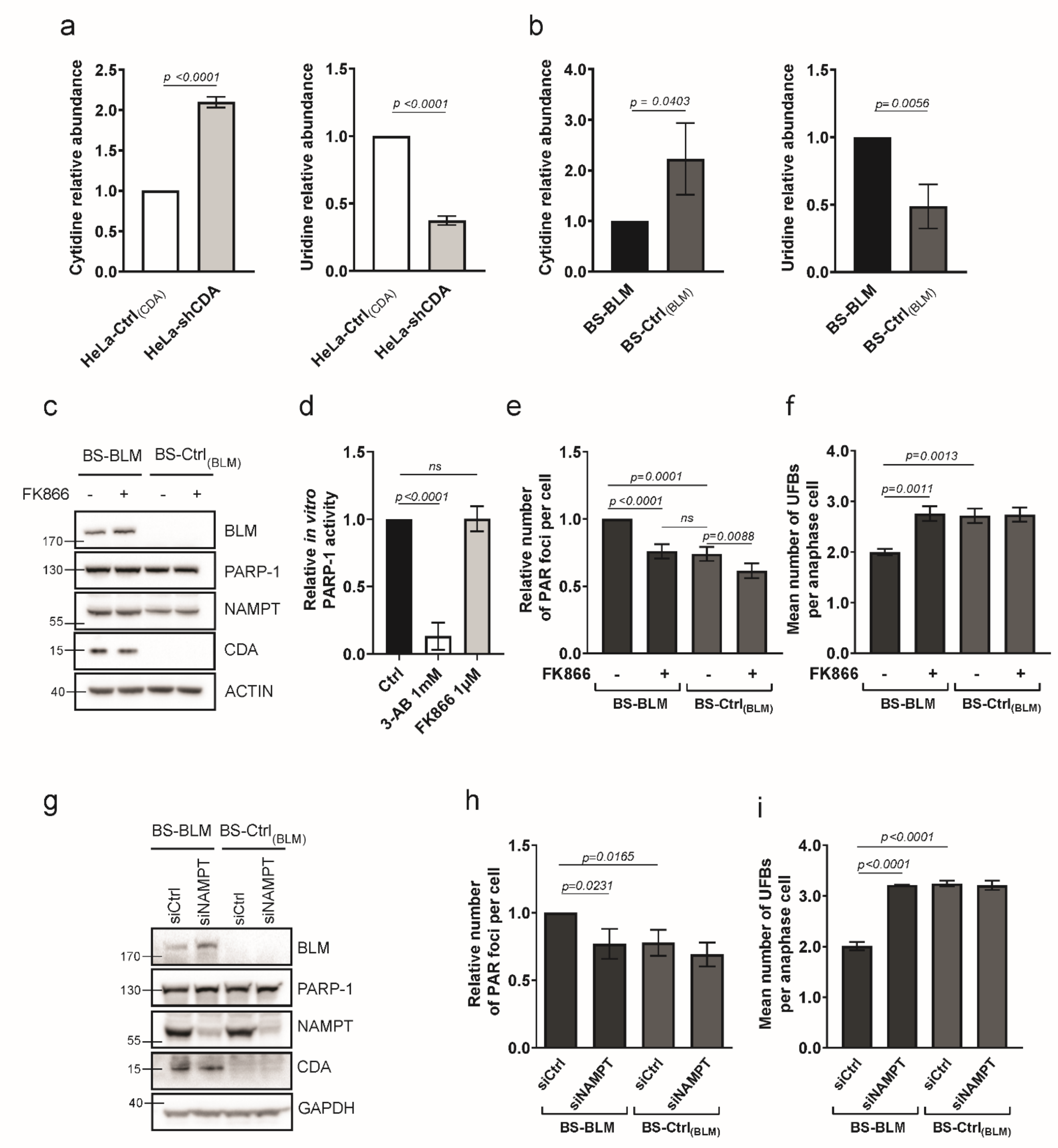

Figure S1. Nucleotide metabolism is altered in CDA-deficient cells and nuclear NAMPT is inhibited by FK866. (a) and **(b)** Graph bars showing the relative abundance of cytidine (left panel) and uridine (right panel) in HeLa-Ctrl$_{(CDA)}$ and HeLa-shCDA cells (a) and in BS-BLM and BS-Ctrl$_{(BLM)}$ cells (b). The data shown are the means ± SD from 3 independent experiments **(c)** BLM, CDA, PARP-1 and NAMPT protein levels were assessed by immunoblotting in BS-

BLM and BS-Ctrl$_{(BLM)}$ cells left untreated or treated with 1 μM FK866 for 10 h. Actin was used as protein loading control. **(d)** *In vitro* analysis of PARP-1 activity in the presence of 1 mM 3-AB or 1 μM FK866. **(e)** Analysis of PAR foci number in BS-BLM and BS-Ctrl$_{(BLM)}$ cells left untreated or treated with 1 μM FK866 for 10 h. The data shown are the means ± SD from four independent experiments (> 500 cells per condition). **(f)** Mean number of UFBs per anaphase cell, for BS-BLM and BS-Ctrl$_{(BLM)}$ cells left untreated or treated with 1 μM FK866 for 10 h. The data shown are the means ± SD from three independent experiments (> 80 anaphase cells per condition). **(g)** BLM, PARP-1, NAMPT and CDA protein levels assessed by immunoblotting in BS-BLM and BS-Ctrl$_{(BLM)}$ cells transiently transfected with the indicated siRNAs. GAPDH was used a protein loading control. **(h)** Analysis of PAR foci number in BS-BLM and BS-Ctrl$_{(BLM)}$ cells transiently transfected with the indicated siRNAs. The data shown are the means ± SD from four independent experiments (> 350 cells per condition). **(i)** Mean number of UFBs per anaphase cell, for BS-BLM and BS-Ctrl$_{(BLM)}$ cell lines transiently transfected with the indicated siRNAs. The data shown are the means ± SD from four independent experiments. The significance of differences was assessed in Student's *t*-tests.

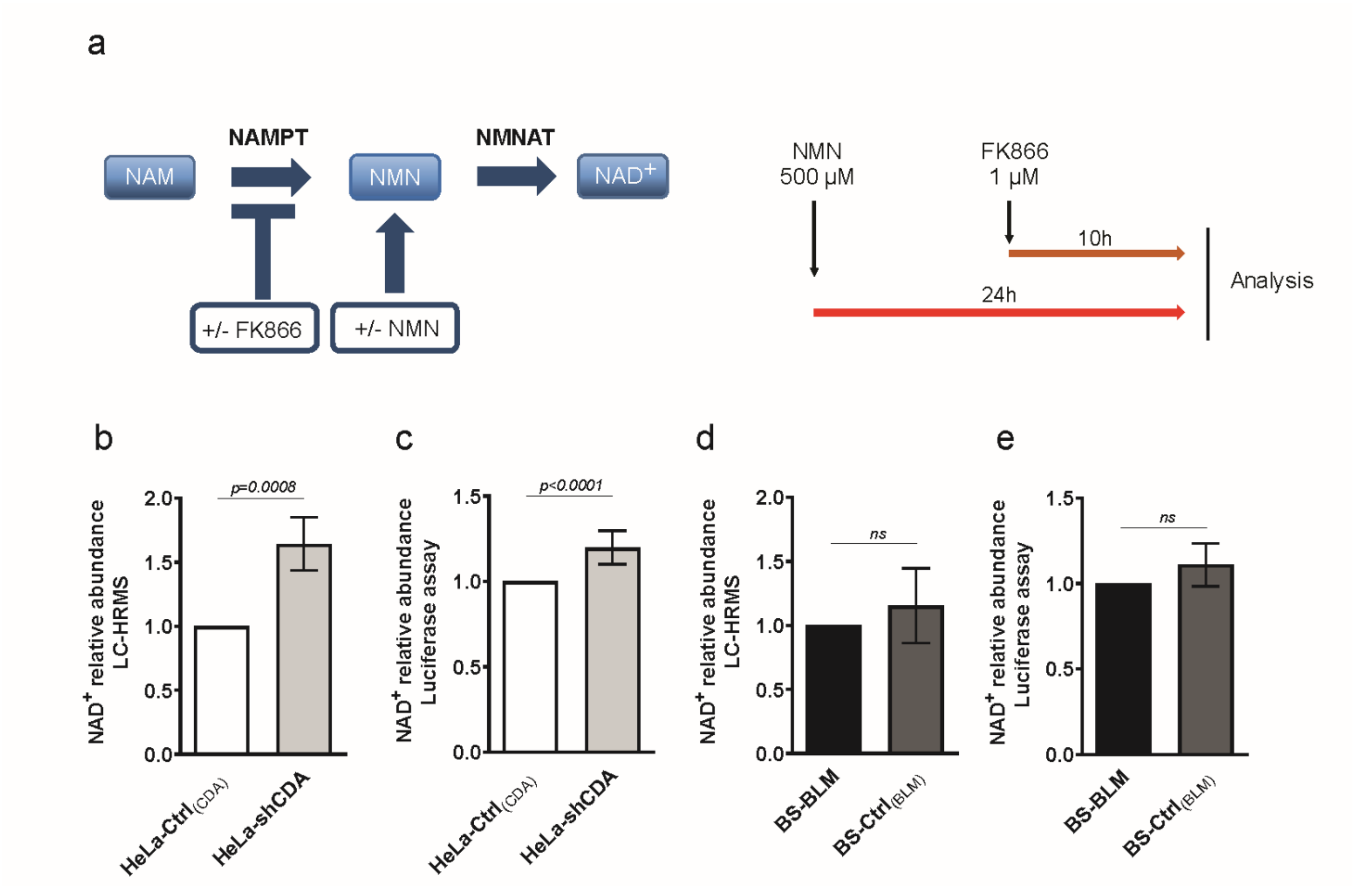

Figure S2. The decrease in basal PARP-1 activity resulting from NAMPT inhibition is independent of NAD$^+$ levels

(a) Schematic representation of the experimental design, showing FK866 and NMN treatments in both CDA-proficient and CDA-deficient cells (left panel). Concentrations and timing of NMN and FK866 treatments in CDA-proficient and in CDA-deficient cells (right panel). NMN was initially added to cell culture medium for a duration of 24 h and FK866 was added to the medium 14 h later for a duration of 10 h before the analysis of NAD$^+$ levels, PAR foci and UFB frequency. **(b)** and **(c)** Analysis of intracellular NAD$^+$ levels in HeLa-Ctrl$_{(CDA)}$ and HeLa-shCDA cells by the LC-HRMS method (b), or in a luciferase assay (c). The data shown are the means $\pm$ SD from 4 and 8 independent experiments, respectively. **(d)** and **(e)** Analysis of intracellular NAD$^+$ levels in BS-BLM and BS-Ctrl$_{(BLM)}$ cell lines by the LC-HRMS method **(d)**, or in a luciferase assay **(e)**. The data shown are the means $\pm$ SD from **3** independent experiments. The significance of differences was assessed in Student's *t*-tests.

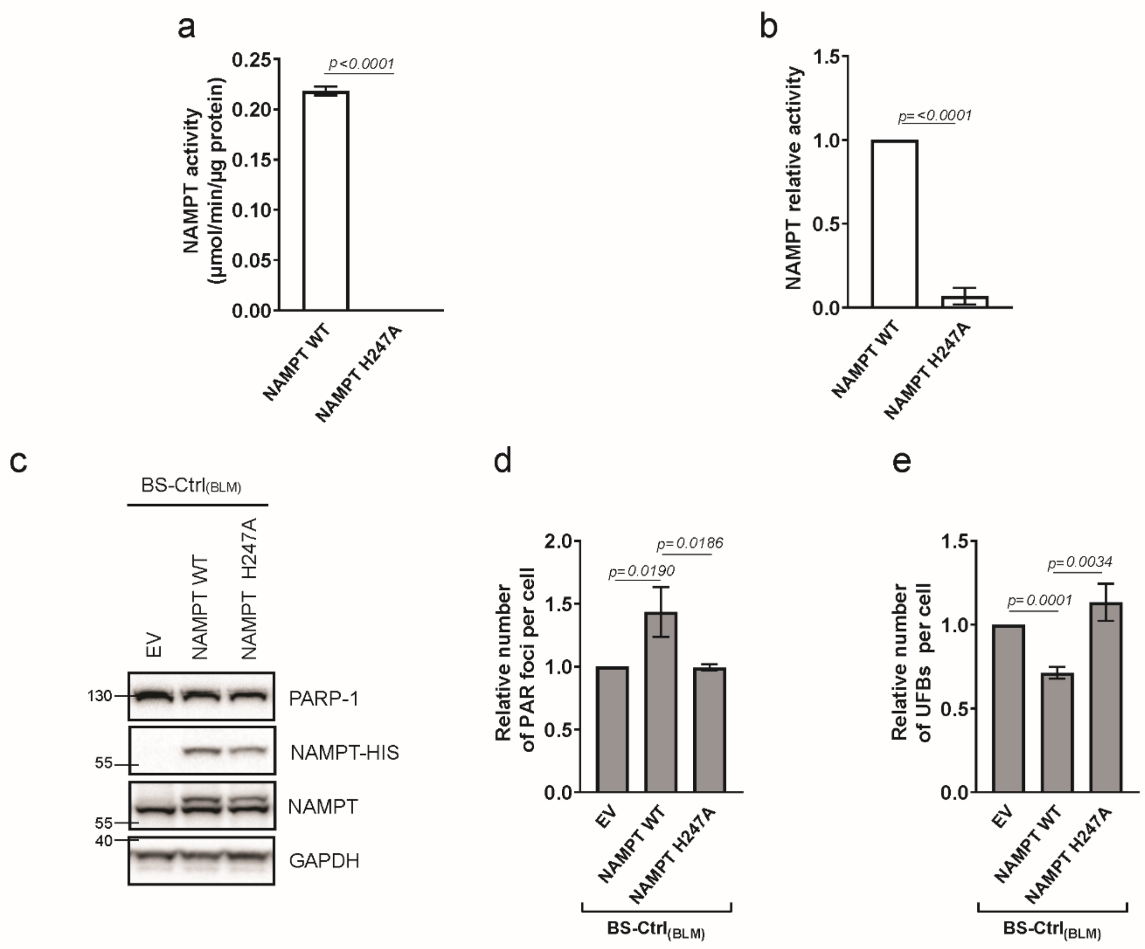

Figure S3. **The low levels of PARP-1 activity in CDA-deficient cells are rescued by the overexpression of wild-type NAMPT. (a)** and **(b)** Measurement of the activity of a wild-type recombinant NAMPT protein (NAMPT WT) and a mutated recombinant NAMPT protein (NAMPT H247A) (a) by LC-HRMS or (b) in an assay measuring the conversion of $^{14}C^{-}$NAM to ^{14}C-NMN. The data shown are means ± SD from three independent experiments. **(c)** PARP-1, NAMPT-HIS, NAMP, and CDA protein levels assessed by immunoblotting in BS-BLM and BS-Ctrl$_{(BLM)}$ cells transiently transfected with EV, NAMPT WT or NAMPT H247A. GAPDH was used as protein loading control. **(d)** Analysis of PAR foci number in BS-BLM and BS-Ctrl$_{(BLM)}$ cells transiently transfected with EV, NAMPT WT or NAMPT H247A. The data shown are means ± SD from three independent experiments (> 570 cells per condition). **(e)** Mean number of UFBs per anaphase cell, for BS-BLM and BS-Ctrl$_{(BLM)}$ cell lines transiently

transfected with EV, NAMPT WT or NAMPT H247A. Errors bars represent means ± SD from three independent experiments (> 110 anaphase cells per condition).

The significance of differences was assessed in Student's *t*-tests.

Supplementary Materials and Methods

Supplementary Table 1

siRNA	Sequence 5' to 3'	Species	Description	Reference
Non-targeting siRNA pool	UGGUUUACAUGUCGACUAA	Human	ONTARGETplus SMART-pool, Dharmacon	[7]
	UGGUUUACAUGUUGUGUGA			
	UGGUUUACAUGUUUUCUGA			
	UGGUUUACAUGUUUUCCUA			
siNAMPT pool	GGUAAGAGUUUCCUGUUA			This work
	CAAAUUGGAUUGAGACUAU			
	UAACUUAGAUGGUCUGGAA			
	CAAGCAAAGUUUAUUCCUA			

Supplementary table 2

**PCR primers**		Sequence 5' to 3'	Reference
**NAMPT – H247A**	**F**	GTTCCAGCAGCAGAAGCCAGTACCATAACAGCT	This work
**NAMPT – H247A**	**R**	AGCTGTTATGGTACTGGCTTCTGCTGCTGGAAC	This work
**NheI**	**F**	TTTGTTTAACTTTAAGAAGGAGATATACAT ATGAATCCTGCGGCAGAAGCCGA	This work
**xhoI**	**R**	ATCTCAATGGTGATGGTGATGGTGCTCGAG ATGATGTGCTGCTTCCAGTC	This work

Figure 1a

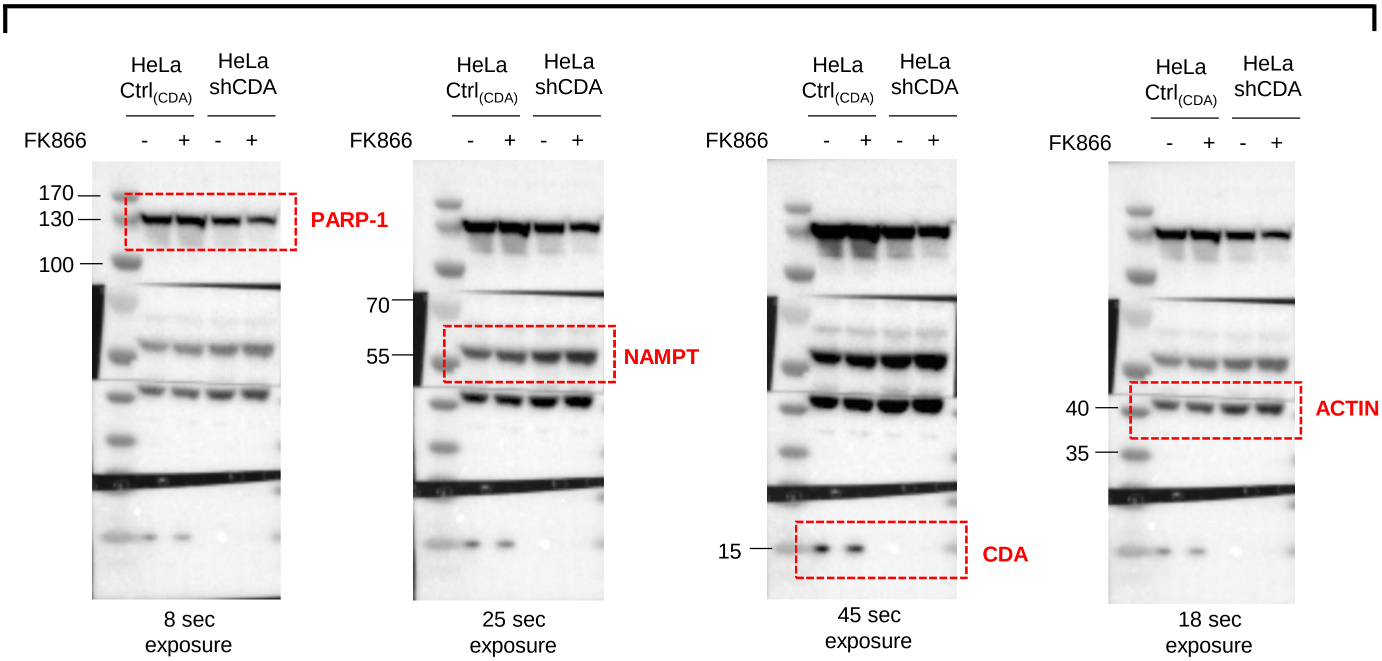

Figure 1f

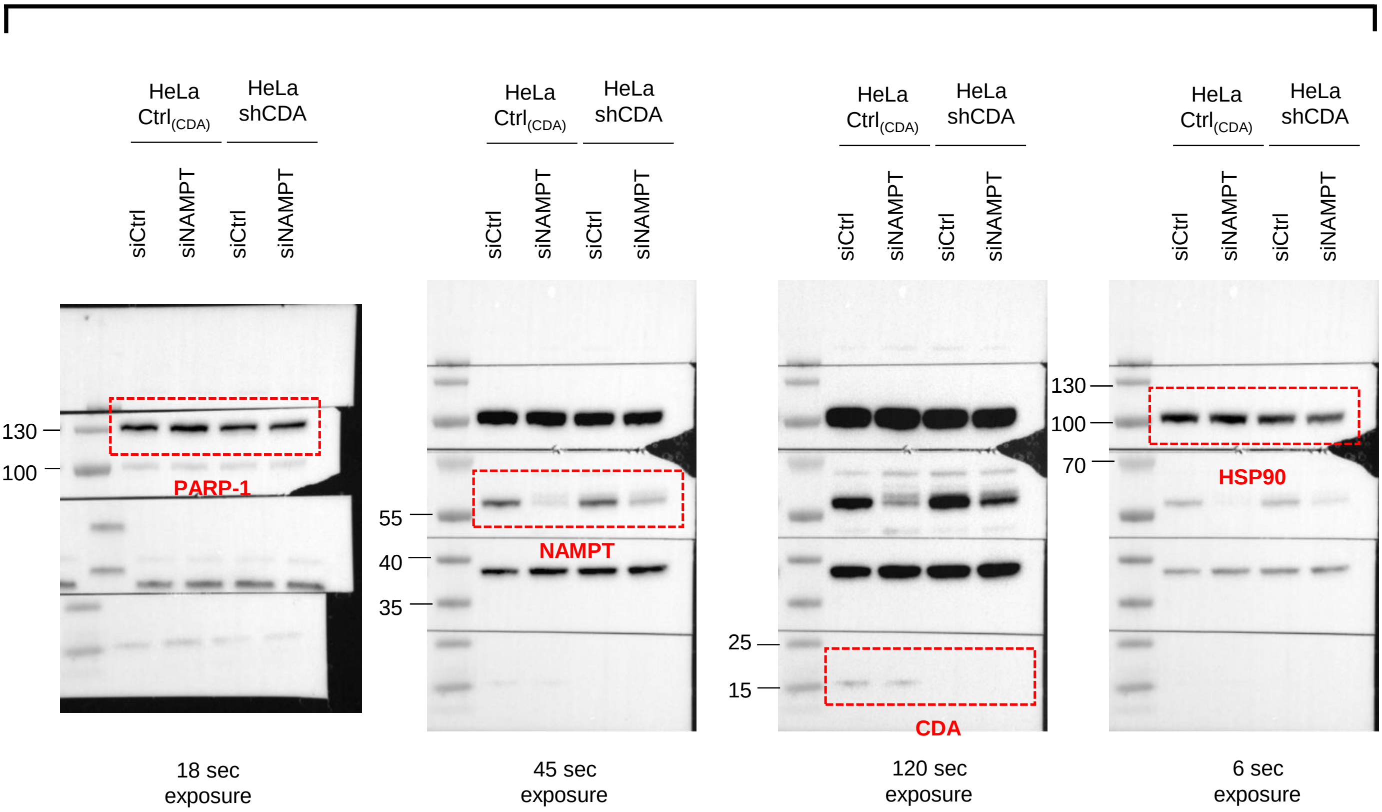

Figure 3c

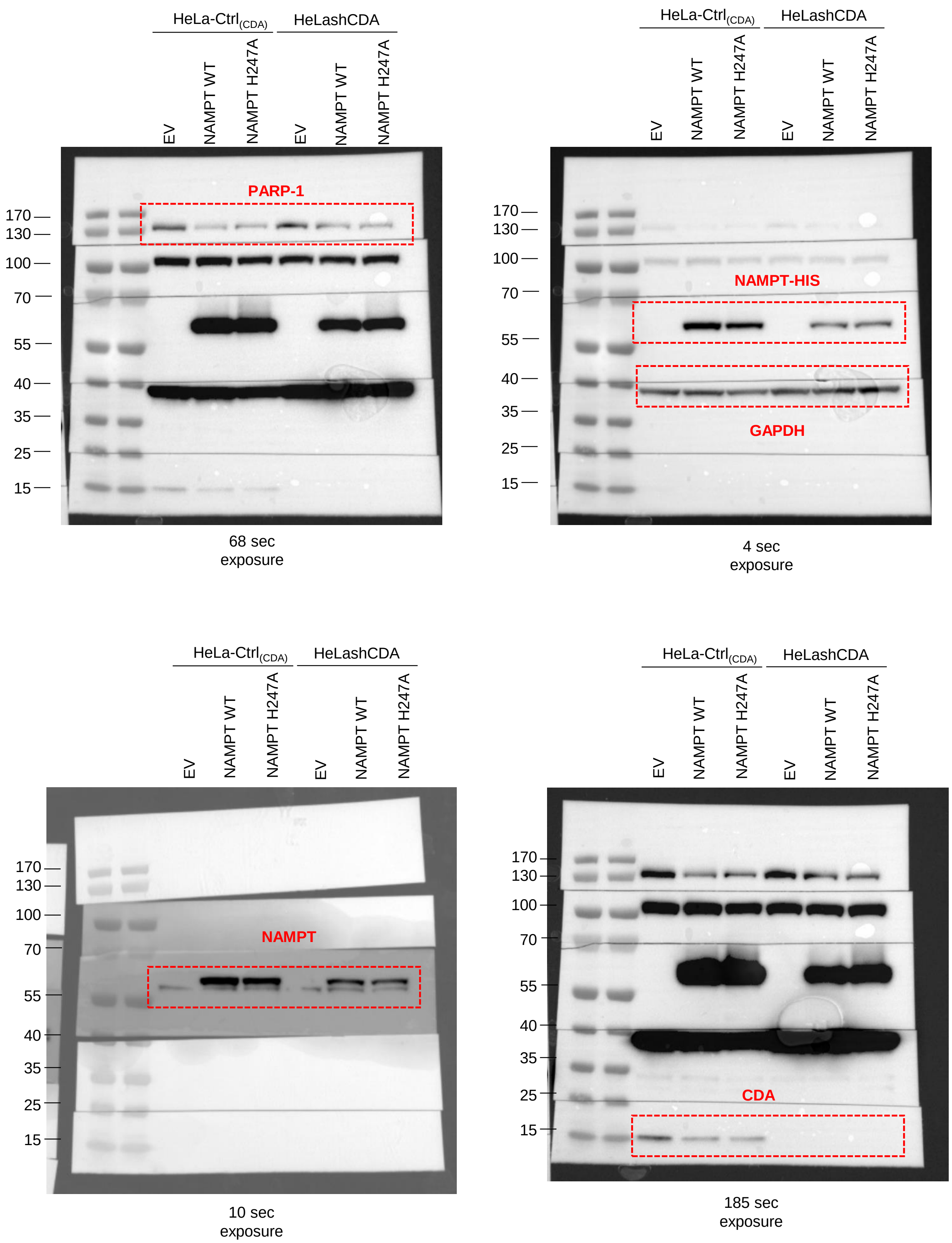

HeLa-Ctrl(CDA)
HeLashCDA
EV
NAMPT WT
NAMPT H247A
EV
NAMPT WT
NAMPT H247A
PARP-1
170
130
100
70
55
40
35
25
15
68 sec exposure
NAMPT-HIS
GAPDH
4 sec exposure
NAMPT
10 sec exposure
CDA
185 sec exposure

Figure S1c

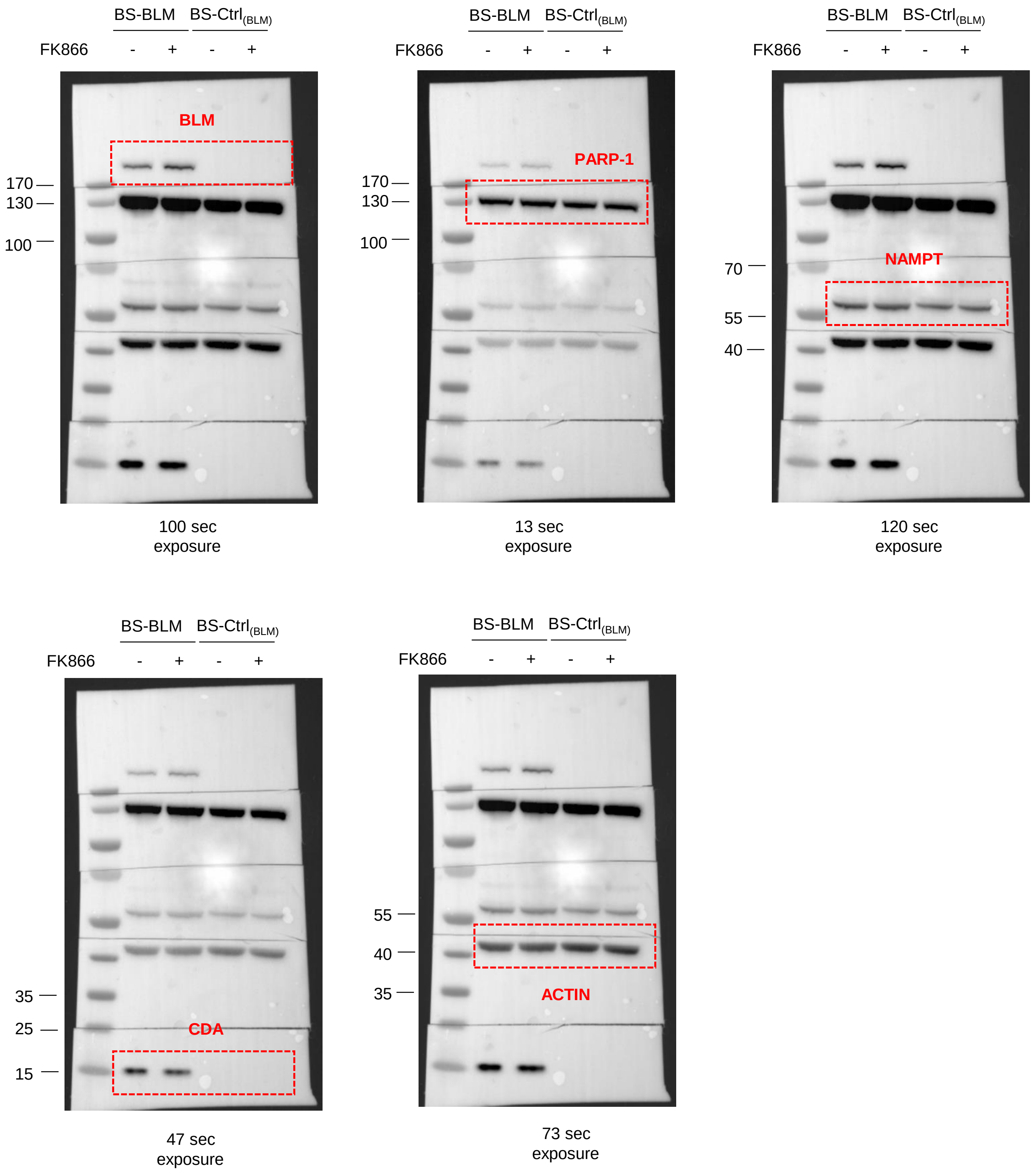
BS-BLM
BS-Ctrl(BLM)
FK866
- + - +
BLM
170
130
100
100 sec exposure
PARP-1
13 sec exposure
NAMPT
70
55
40
120 sec exposure
CDA
35
25
15
47 sec exposure
ACTIN
73 sec exposure

Figure S1g

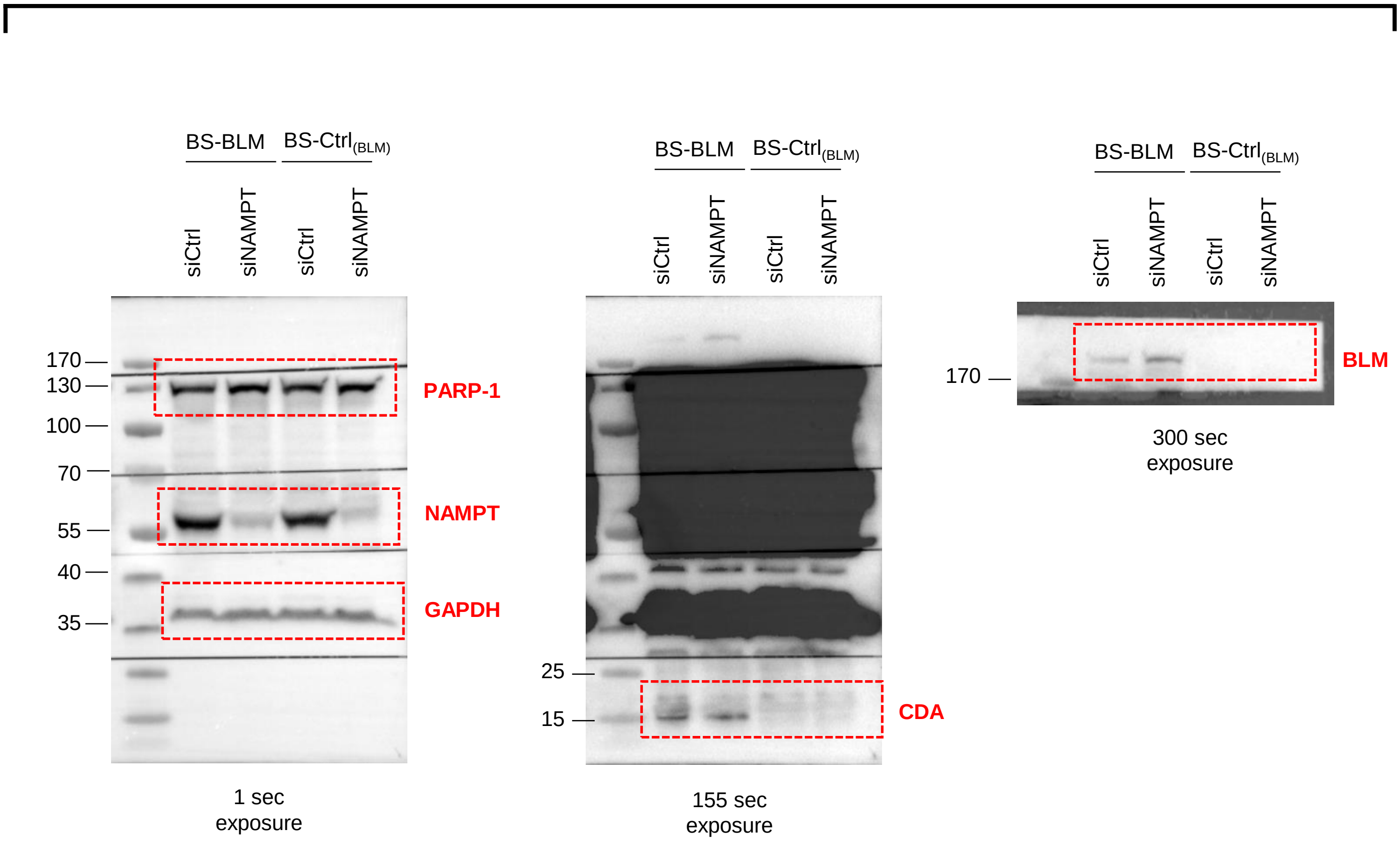

BS-BLM
BS-Ctrl(BLM)
siCtrl
siNAMPT
siCtrl
siNAMPT
170
130
100
70
55
40
35
PARP-1
NAMPT
GAPDH
1 sec exposure
BS-BLM
BS-Ctrl(BLM)
siCtrl
siNAMPT
siCtrl
siNAMPT
25
15
CDA
155 sec exposure
BS-BLM
BS-Ctrl(BLM)
siCtrl
siNAMPT
siCtrl
siNAMPT
170
BLM
300 sec exposure

Figure S3c

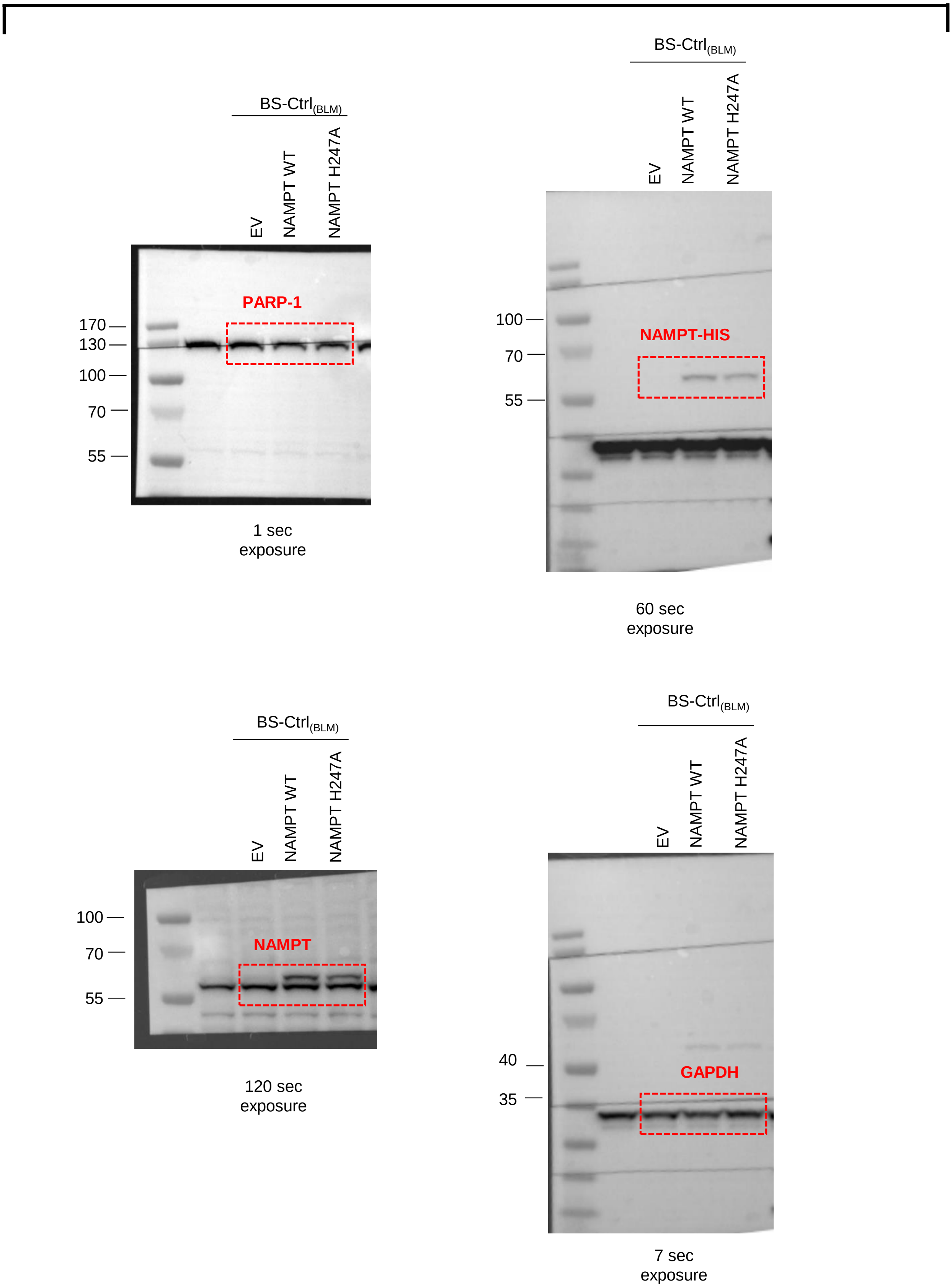
BS-Ctrl(BLM)
EV
NAMPT WT
NAMPT H247A
PARP-1
170
130
100
70
55
1 sec
exposure
BS-Ctrl(BLM)
EV
NAMPT WT
NAMPT H247A
NAMPT-HIS
100
70
55
60 sec
exposure
BS-Ctrl(BLM)
EV
NAMPT WT
NAMPT H247A
NAMPT
100
70
55
120 sec
exposure
BS-Ctrl(BLM)
EV
NAMPT WT
NAMPT H247A
GAPDH
40
35
7 sec
exposure
